# Fault-Level Grading of Photovoltaic Cells Employing Lightweight Deep Learning Models

**DOI:** 10.1155/2023/2663150

**Published:** 2023-02-07

**Authors:** Ikramullah Khosa, Abdur Rahman, Khurram Ali, Jahanzeb Akhtar, Ammar Armghan, Jehangir Arshad, Melkamu Deressa Amentie

**Affiliations:** ^1^Department of Electrical and Computer Engineering, COMSATS University Islamabad, Lahore Campus, Islamabad 54000, Pakistan; ^2^Department of Electrical Engineering, College of Engineering, Jouf University, Sakaka 72388, Saudi Arabia; ^3^Assosa University, Assosa 5220, Ethiopia

## Abstract

The deployment of photovoltaic (PV) cells as a renewable energy resource has been boosted recently, which enhanced the need to develop an automatic and swift fault detection system for PV cells. Prior to isolation for repair or replacement, it is critical to judge the level of the fault that occurred in the PV cell. The aim of this research study is the fault-level grading of PV cells employing deep neural network models. The experiment is carried out using a publically available dataset of 2,624 electroluminescence images of PV cells, which are labeled with four distinct defect probabilities defined as the defect levels. The deep architectures of the classical artificial neural networks are developed while employing hand-crafted texture features extracted from the EL image data. Moreover, optimized architectures of the convolutional neural network are developed with a specific emphasis on lightweight models for real-time processing. The experiments are performed for two-way binary classification and multiclass classification. For the first binary categorization, the proposed CNN model outperformed the state-of-the-art solution with a margin of 1.3% in accuracy with a significant 50% less computational complexity. In the second binary classification task, the CPU-based proposed model outperformed the GPU-based solution with a margin of 0.9% accuracy with an 8× lighter architecture. Finally, the multiclass categorization of PV cells is performed and the state-of-the-art results with 83.5% accuracy are achieved. The proposed models offer a lightweight, efficient, and computationally cheaper CPU-based solution for the real-time fault-level categorization of PV cells.

## 1. Introduction

**Figure 1:**
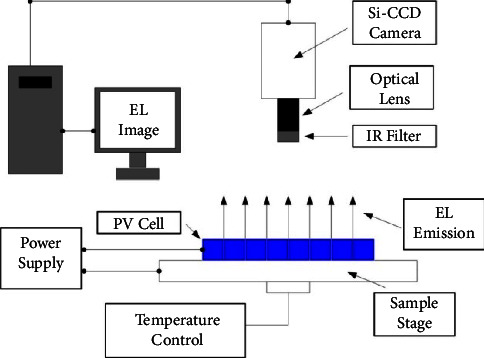
Fault detection in PV cells via electroluminescence imaging.

With the beginning of 21st century, stimulation of improving energy-efficient policies increased the public interest towards renewable energy, especially the solar energy, since it is noiseless and pollution-free. This interest opened the gates for research studies in achieving optimal performance for solar energy systems. Energy systems, in general, are of two types: active systems and passive systems [1]. Passive systems do not consume energy; rather they convert energy from one form to another. Photovoltaic (PV) systems are purely passive systems as the electrical energy is generated directly from semiconductors by the photovoltaic effect. With the passage of time, different kinds of faults may occur decreasing the PV cells efficiency such as hotspot fault, diode fault, junction box fault, ground fault, arc fault, and line-line fault [2]. Such faults may rise due to glass breaking, oxidization, delamination of cells, and bubbling [2]. Apart from reducing efficiency, there is a risk of fire as well. For detection and diagnosis of such faults, there are several methods in practice including signal processing, statistical approaches, current-voltage curve analysis, power loss analysis, and machine learning-based techniques [3]. For detection of fault, a comparison between reference and observed measurement is made. To maintain the PV system's efficiency, the faults must be detected in the first place followed by isolation or maintenance of the faulty cell. To detect the faults such as microcracks in PV systems, several techniques can be found in the literature [4]. One among them is Laser Beam Induced Current (LBIC). It is an optical way to observe microcracks in PV cells [5, 6]. An AC laser beam of wavelength ranging from 638 nm to 850 nm is produced by modulating the electric current through the laser diode and directed on the photosensitive device. This causes direct current (DC) to flow through the semiconductor. Large current variation by changing PV cell position indicates the presence of the defect [7–9]. Another technique, Electron beam induced current (EBIC) is the semiconductor-based analysis technique in which the current is induced in the sample and used as a triggering signal for image generation. The image highlights the local defects of the PV cell. Most of the electronic beam techniques are performed using scanning electron microscope (SEM) [10]. Photoluminescence (PL) imaging is another method where electrons get excited in the conduction band after the photon is absorbed. It causes recombination of the electron hole pair. The image is captured through CCD camera. The electroluminescence (EL) imaging method is also used for detection of microcracks of the wafers and solar cells by employing luminescence imaging. EL is a form of luminescence in which electrons get excited in the conduction band when electric current in forward bias mode is passed through it. The excitation of electrons emits infrared radiation at wavelength ranging 950–1250 nm. The image of solar cell is then captured through charge-coupled device (CCD) cameras [10]. The defective or disconnected PV cell appears darker [11]. The defect can be conveniently located through visualization. The EL imaging phenomenon is shown in Figure 1. The difference between EL and PL is that, the PL technique involves the excitation of electrons using laser light instead of electric current [12]. The EL imaging technique works for finished PV cells, whereas the PL imaging technique is used for both wafers and solar cells.

The convolutional neural networks (CNNs) have been successfully employed for pattern recognition tasks. However, in literature, only a few studies exist involving CNN for EL image data classification, particularly the defect-level classification. Most of them focused on the binary classification problem where the GPU-based computationally expensive solutions were proposed. In fact, merely a couple of studies proposed CPU-based cheaper and real-time solutions. In this paper, we aim to develop the lightweight CNN-based models suitable for CPU machines for real time processing. The contributions of this paper are summarized as follows:

- The deep learning capability of fully connected neural network is experimented with hand-crafted features for fault-level classification of PV cells. Data augmentation is performed to balance the class representation and both preaugmentation and postaugmentation results are presented.
- The two-way binary classification is performed by segregating the data in two separate ways; hence, the two independent results of binary classification are presented.
- A customized, simpler, and computationally efficient CNN architecture is developed ensuring real-time classification of EL image data. The proposed CNN model has 50% less parameters than the state-of-the-art CNN-based solution.
- The proposed CNN model achieved state-of-the-art results with a 23× shorter training time. Moreover, the state-of-the-art result of multiclassification of the EL image data is presented.

The rest of the paper is organized as follows: Section 2 presents the related work, the detail of dataset is provided in Section 3, the methodology is explained in Section 4, performance evaluation strategy and the metrics are presented in Section 5, Section 6 includes results and discussion, Section 7 presents a detailed comparative analysis, and finally, conclusion is added in Section 8.

## 2. Related Work

One of the basic approaches for fault detection is the comparison of observed output power with the reference power. The difference higher than the defined threshold indicates the presence of fault [13, 14]. A study presented the application of Kalman filter for the prediction of power output [15]. The noisy measurements were taken as input and given to the physical underlying model, which produced an output value with the highest probability. The output was then used to locate the faults within the measurements of voltage, current, and the power. Artificial intelligence (AI)-based techniques have also been used for fault detection. Bayesian and fuzzy logic algorithms were tested to determine the PV cell output [16]. Both supervised and unsupervised machine learning techniques have been employed for PV system fault diagnosis such as k-nearest neighbor, decision tree, and support vector machine (SVM) [17]. In a study, an artificial neural network (ANN) model was developed to locate the short circuit (SC) in PV cell [18]. In another study, Bayesian network (BN) was used to describe the causes of the detected faults [19]. Another research claimed the error rate of 0.35–0.55 by combining two approaches: SVM and *k*-nearest neighbor for the detection of PV faults [20]. Mohamed and Nassar presented an ANN-based solution for the diagnosis and repairing of PV systems [21]. The abovementioned studies involved machine learning techniques for fault detection and diagnosis. However, the features used in those studies were the measured readings of current, voltage, and power, which involve the tedious manual inspection and record.

Considering the physical inspection scenario, in most of the cases, PV systems are placed at elevated places making it difficult to locate the fault and isolate the faulty cell [22]. An unmanned arial vehicle (UAV) equipped with thermal camera may be a solution for fault detection in such a scenario [23]. The images captured with thermal camera unveil the location of the fault in solar cells very conveniently. Recent literature shows the usage of thermography for the detection as well as classification of PV faults. However, it has its own limitations [24–26]. The infrared (IR) imaging has also been carried out for fault detection in solar cells [9, 27]. However, it is challenging to locate the exact location of the fault and identify microcracks in the infrared images due to its relatively low resolution. The author concluded that a hot region in IR images may result as a false positive. The EL imaging, as mentioned earlier, is the technique to identify the faults in PV cells, which involves capturing the infrared energy emitted from the cell in the form of gray-scale image. The resultant EL image provides better resolution than an IR image [28, 29]. Various studies have been carried out for fault detection in PV cells, but a few considered EL imaging. A study presented Fourier image reconstruction technique for fault detection in EL images [30]. However, the authors considered limited defects including finger interruptions, small cracks, and breaks. Moreover, it was a complex detection method due to shape assumptions. Another study [31] used independent component analysis (ICA) for defect detection; however, finger interruption and cracks were considered equal defects. In another study, Stromer presented vesselness algorithm for crack segmentation. However, the cracks larger than 20 mm in size were considered [32]. Recently, deep learning and convolutional neural networks (CNNs) have been employed for defect detection in PV modules. In a study [33], automatic inspection of PV module was presented using deep learning, however, only visible defects were considered. Similarly, multispectral CNN was proposed for visible fault detection [34]. A recent study presented the fault classification in electroluminescence images including the categories as defect-free, microcrack, break, and finger-interruption [35]. The dataset was collected from a private company as well as from public domain. The general adversarial network (GAN) was used for data augmentation and pretrained CNN models were used for defect classification, originally trained for ImageNet challenge [36]. Another study used CNN model for defect categorization in PV cells [37]. However, the IR image data was utilized for this purpose.

Apart from defect detection or categorization, recently, studies have been conducted on detection of the level of the defect. A study presented deep learning approach for defect-level classification in PV cells using EL images [38]. For this purpose, a public dataset of EL images labeled with four distinct defect levels as classes was used [39]. The author used VGG-19 pretrained CNN model and tuned it via transfer learning. The model produced 88.4% accuracy for binary classification; however, the experiment was performed on the graphics processing unit (GPU)-based machine, making it a computationally expensive solution. The authors also performed SVM-based classification for real-time processing and achieved 82.4% accuracy. Another recent study proposed light CNN architecture for defect level binary classification of the same EL image data [40]. The authors considered the VGG-11 as the initial CNN architecture and further simplified it to obtain the optimal and light architecture for the classification task. The authors claimed 93.02% classification accuracy; however, it was not mentioned how the original four target classes ended up into two classes to perform binary classification. The data augmentation was also performed but there is no mention of the total samples, as well as the evidence of balancing the classes after performing the augmentation.

Concretely, the analysis and classification of defects as well as fault-level classification in PV images in real time still demand further research in many aspects such as increasing data volume via efficient data augmentation methods; the estimation of simpler and optimized machine learning algorithm to enhance robustness; and to estimate a lighter network for achieving real-time processing. In addition, being four-labeled categories of the publicly available EL image data [39], there is no evidence of multiclass classification in the literature. Therefore, there is a need to develop a system for multilevel categorization as well.

## 3. The Data Set

For this research, a publicly available dataset of EL images is used [39]. It consists of 2,624 image samples of healthy as well as faulty PV cells. Each sample is an 8-bit gray-scale image with a resolution of 300 × 300 pixels. These image samples were originally extracted at cell level from mono and poly-crystalline PV modules, and were normalized with respect to perspective and size. The details of the dataset are summarized in Table 1.

The original images were initially analyzed by the human experts based on working condition of the cell, and labels were assigned in terms of defect probability. There are four distinct classes labeled with defect probability 0.0, 0.33, 0.66, and 1.0, where defect probability 0.0 represents the full healthy cell; defect probability 0.33 characterizes less faulty cell; 0.66 represents medium faulty and defect probability, and 1.0 denotes full faulty cell. Concretely, the defect probability represents the defect-level of the PV cell.

**Figure 2:**
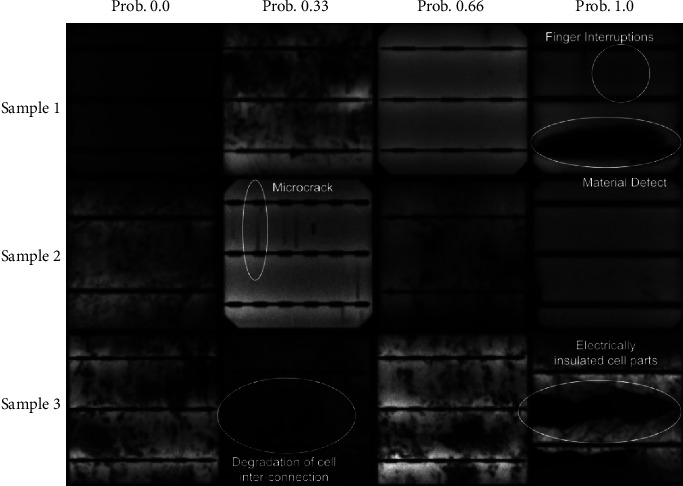
Few samples from the EL image dataset with distinct defect probabilities shown with different kinds of defects.

Few samples from the dataset belonging to individual categories are shown in Figure 2. There are different types of labeled defects including microcracks, material defect, finger interruptions, and fracture interconnect which affected the PV cell efficiency. Although, the EL image dataset consists of image samples with four class labels; however, the number of images belonging to each class are not the same. The maximum number of samples belongs to healthy class (defect probability 0.0), whereas the least representation is of the class with defect probability 0.66. The count of original classwise samples in the dataset is given in Table 2.

## 4. Methodology

### 4.1. Artificial Neural Network Model

Among the data-driven approaches for pattern recognition and classification applications, ANN has been successfully used in the last couple of decades. Customized architectures of feed-forward neural network can be estimated to accommodate the complex nature of the input-output relationship of the data. In this work, deep ANN architectures are employed. For the classification of EL image data, the ANN architectures are estimated starting with single hidden layer and then extended up to multiple hidden layers until optimized. The size of each hidden layer is also estimated in the process by observing the cross-validation error. The Levenberg–Marquardt (LM) algorithm is used for training the network with zero mean square error (MSE) as the convergence criteria. The description of the final estimated architectures is added in the results section.

### 4.2. Hand-Crafted Features

The ANN requires features to be fed with. There are several kinds of features which may be considered, including polynomial features [41–43]. However, we opted for two kinds of popular and widely used hand-crafted features: gray-level co-occurrence matrix (GLCM) features and local binary pattern (LBP) features.

#### 4.2.1. Gray Level Co-Occurrence Matrix

A GLCM represents spatially joint probabilities of pixel intensities in the image. The features computed from GLCM are classic yet effective and provide the texture analysis of the image, originally proposed by Haralick et al. [44]. A total of the following 22 features from each of four GLCMs computed at angles 0, 45, 90, and 135 degrees are computed [45]: autocorrelation 1, contrast, autocorrelation 2, cross-correlation, cluster prominence, cluster shade, dissimilarity, energy, entropy, homogeneity, maximum probability, sum of squares, sum average, sum variance, sum entropy, difference variance, difference entropy, information measure of correlation 1, information measure of correlation 2, inverse difference, inverse difference normalized, and inverse difference moment normalized. A total of 88 features were extracted from each image.

#### 4.2.2. Local Binary Patterns

Local binary patterns (LBP) as features have also been widely used for the applications of pattern recognition and computer vision. The simplest LBP feature vector is generated as per the following steps:

- Dividing the window to be examined into cells of 9 × 9 pixels per cell.
- Comparing each neighboring pixel with the central pixel
- Assigning it value 0 if it is less than the central pixel value, otherwise 1. This provides an 8-bit binary number.
- Computing the histogram of the cell for the frequency of every number occurring.
- Normalizing the histogram.
- Concatenating the normalized histograms of all the cells.

From each of the image, a 59-dimensional LBP feature vector was computed.

### 4.3. Customized Convolutional Neural Network

CNNs have been used successfully in recent past for several applications from simple visual recognition tasks up to vehicle's autonomous driving systems. A CNN consists of convolutional layers, pooling layers, activation functions, and fully connected (FC) layers. The convolutional layer plays a vital role since it extracts the features from the images. The first convolutional layer is connected to the raw pixels, extracting low-level features like edges, where the next layer gets medium-level information, and subsequently the next layers extract high-level features. The pooling layer is employed to reduce the size of the learned features by ignoring less important information. The FC layer is similar to the one in ANN, where each neuron from the previous layer is connected to every neuron in the current layer. The number of neurons in the output layer is kept equal to the number of output labels. By customized, it means that the network is built from scratch. The architecture is selected by increasing the size and recording the classification results on training data. The details of the architectures and the classification tasks are described in the results section.

### 4.4. Data Augmentation

The EL image dataset has four distinct classes. It can be observed from Table 3 that the representation of full faulty class (defect probability 1.0) is almost 50% of that of representation of healthy class (defect probability 0.0). It can also be seen that there is significantly a small representation of the medium faulty class (defect probability 0.33). The least representation among all is of medium faulty class (defect probability 0.66). Since the machine learning techniques are data-driven approaches, therefore, for any dataset, the classifier is inherently biased towards the target class having the largest number of samples. Therefore, to assure the unbiased learning of the classifier, it is important to balance the target classes before the model training. This is normally done using data augmentation when the acquisition of new data is not an easy process. It is also extremely important that the augmentation to be applied to the training data only, while the test data be separated beforehand. In other words, the classifier should be trained with the training data where it may have augmented samples to balance all the target classes; however, the test data should be original without any augmented sample.

Initially, the original data is randomized and divided into training, validation, and testing at 70%, 15%, and 15%, respectively. At this stage, both validation data and test datasets have each 226, 45, 16, and 107 images for healthy, less faulty, medium faulty, and full faulty classes, where these sets were separated for later use. Next, the training data is used for augmentation to balance the classes so that the unbiased training of the classifier can be made sure. For augmentation, affine transformation is performed, including horizontal and vertical translation with ±10 pixels, image rotation at ±90°, horizontal and vertical flip, and the intensity transformation with a variation of ±5% in the original pixel intensity. The number of samples in the training data before augmentation, belonging to healthy, less faulty, medium faulty, and full faulty classes were 1056, 205, 74, and 501, respectively. The postaugmentation training data size is limited to 6,000 samples in total, with 1,500 samples per category. The augmented samples have an equal distribution of four types of transformations. Since the class representation is kept equal after augmentation, the postaugmented training data has the maximum number of augmented samples (1500 − 74 = 1426 augmented samples) for the medium faulty class, as it had the least number of training samples (74 samples) before augmentation. Consequently, the healthy class has the least augmented samples (1500 − 1056 = 444 augmented samples), and the medium faulty class has the most augmented samples (1, 426) in the postaugmented training data. For evaluation of results, both preaugmented (the original data) and postaugmented data are used separately, and individual classifiers are trained.

## 5. Evaluation Strategy and Metrics

As mentioned previously in the data augmentation section, the data is divided into training, validation, and test sets at 70%, 15%, and 15% respectively. For ANN classifier, training data is used for training the model, validation data to optimize it, and the test data for results evaluation. Therefore, only test data containing 15% of unseen original samples is used to evaluate the results of the deep ANN classifier. For CNN model, training data is used for training, and the test data is used for result evaluation. The classification of PV cells is performed three-way: two separate results are recorded for binary classification, and one for multiclass classification. The classifications tasks are defined as follows:

- *Binary Classification*. In this classification task, the data of two classes are considered only: healthy class and full faulty class. The data of the remaining two classes are not used in this case.
- *Binary Classification with* 0.5 *as Threshold*. In this classification task, the data of all classes are used but represented with two labels only: healthy and full faulty. For this purpose, samples of healthy class and less faulty class are combined and labeled as healthy class. The samples of medium faulty and full faulty class are combined and labeled as full faulty class. In other words, defect probability of 0.5 is used as threshold to convert four classes into two categories, which is why this task is defined as binary classification with 0.5 as threshold.
- *Multiclass Classification*. In this task, the data are classified as per their original class label being healthy, less faulty, medium faulty, or full faulty. Concretely, all four classes are considered for multiclass classification.

**Figure 3:**
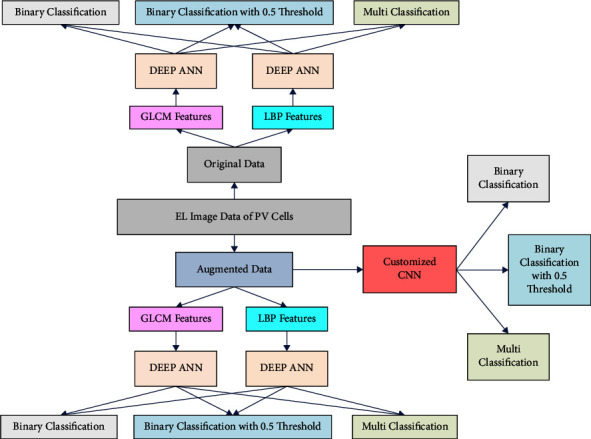
Block diagram of overall methodology of the PV dataset classification.

The block diagram of the overall methodology is shown in Figure 3. For each of the classification tasks, the estimated optimal network architecture, the choice of hyper parameters, and the corresponding results are discussed in the following section. As described earlier, the results are calculated separately on preaugmented (original) data as well as postaugmented data.

### 5.1. Hardware Details

The experiment is performed on a laptop system with the following hardware specifications: Intel core i3 CPU with 2.4 GHz clock speed and 2 GB RAM. The software used was MATLAB 2018b in Windows 10 environment.

For the evaluation of results, the confusion matrices with true positives, false positives, true negatives, and false negatives are shown. The results of classwise accuracy and the overall classification accuracy are also presented. Moreover, the receiver operative characteristic (ROC) curves along with the area under the curve (AUC) are presented.

## 6. Results and Discussion

### 6.1. Deep Feed-Forward Neural Network Results

In this section, the test data results for binary as well as multiclass classification using deep architectures of feed-forward artificial neural network are presented. Hand-crafted features i.e., GLCM and LBP are used for data representation, and therefore, fed to the ANN architectures as features. It is worthy to mention that for each task, a number of network architectures were tested; however, the architectures of only the best models are described in the following subsections.

#### 6.1.1. Binary Classification Using Original Data

For binary classification with original (preaugment) data employing GLCM and LBP features, the network architecture is optimized by varying the number of hidden layers as well as the size of hidden layers. The input layer is fed with features, while the output layer has two neurons. For GLCM features, the final optimized network architecture has five hidden layers with [30-30-20-20-10] neurons, respectively. Similarly for LBP features, the optimized network consists of three hidden layers with 30 neurons in each hidden layer. The test data classification results using the GLCM and the LBP features are presented in Table 3 (a) and (b), respectively. It can be observed that the network with LBP features achieved 8.5% higher accuracy overall than the network fed with GLCM features. A similar pattern can be seen in the case of individual class accuracy.

#### 6.1.2. Binary Classification Using Augmented Data

**Figure 4:**
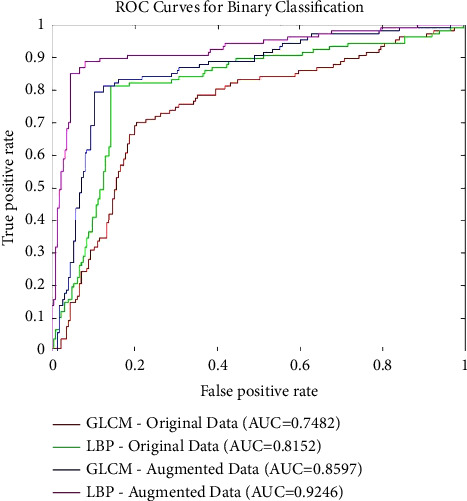
Receiver operative characteristic curve analysis of binary classification results employing ANN with hand-crafted features for preaugmentation and postaugmentation data.

After augmentation, the network is trained using 1,500 images from each class. The estimated optimized architecture for this set of data is composed of three hidden layers both for the GLCM and LBF cases with 30 and 10 neurons in each layer, respectively. It is evident that the architectures using augmented data are shallower as compared to the ones estimated previously for preaugmented data. This is because of improved learning of the network due to its large and balanced representation of classes. The accuracy is improved by 10% for GLCM features, and 7.8% for LBP features with augmented data, as shown in Table 4. The maximum overall accuracy for binary classification achieved is 92.2% by ANN fed with LBP features. Overall, the LBP features combined with the optimized architecture of deep ANN produce the best accuracy among the four cases (original data with LBP and GLCM; augmented data with LBP and GLCM) of binary classification.  Figure 4 presents the ROC curves for binary classification results obtained in the discussed four cases. The AUC value for each case is also shown. The highest AUC can be observed for ANN classifier fed with LBP features when augmented data are used.

#### 6.1.3. Binary Classification with 0.5 as Threshold Using Original Data

For this second kind of binary classification with original data, the optimized network architecture has five hidden layers with sizes [30-30-20-20-10] and three hidden layers with sizes [30-30-30] for GLCM and LBP features, respectively. The test data classification results are shown in Table 5. On comparing these results with the results of binary classification in Section 6.1.1 (where only two classes were used: healthy and full faulty of the original data) in context of features' significance, the current results are contrarily better for GLCM features and worse for LBP features. The reason is the merger of four classes into two classes, which increases the complexity of the problem and affected the classifier's performance. After data division using a 0.5 defect probability, the GLCMs proved to have better hand-crafted features than LBPs.

#### 6.1.4. Binary Classification with 0.5 as Threshold Using Augmented Data

After augmentation, the optimized architectures ended up having three hidden layers with sizes [30-20-10] and [30-30-30] for GLCM and LBP features, respectively. It can be observed once again (as discussed in Section 6.1.2) that after augmentation, the results are improved for both feature kinds, where the GLCM produced better results than the LBP, as shown in Table 6. By comparing the previous similar scenario in Section 6.1.2 (postaugmentation binary classification results shown in Table 4), the current results are inferior due to the fact of merging the four classes into two.

**Figure 5:**
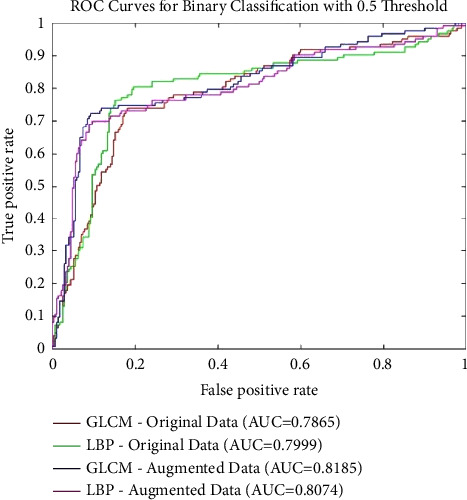
Receiver operative characteristic curve analysis of binary classification results with 0.5 as threshold employing ANN with hand-crafted features for preaugmentation and postaugmentation data.

Among all four cases discussed above in subsections 5.1, the best results are obtained using augmented data with GLCM features, where the overall achieved accuracy is recorded at 84.8%. Among the GLCM and LBP feature choices, the accuracy for the defective class is the same; however, the accuracy using GLCM features has increased for the healthy class, leading to an increase in overall accuracy in the case of GLCM features, as shown in Table 6. The results of LBP features are improved after augmentation.  Figure 5 presents the ROC curves for binary classification results with 0.5 threshold obtained in the above discussed four cases. It can be noted that the value for AUC for all four cases is similar; however, the best performance can be observed for GLCM features using augmented data.

#### 6.1.5. Multiclass Classification Using Original Data

Here the results of multiclass classification for four classes using the original data with deep ANN are presented. The optimized architecture ended up having five hidden layers with [30-30-20-20-10] hidden neurons and three hidden layers with 30 neurons per hidden layer for GLCM and LBP features, respectively. The LBP features showed 7.8% higher accuracy than the GLCM features, as shown in Table 7. If we look at the results, it can be observed that the network fed with LBP features was able to predict 46.7% of samples from the less faulty class correctly, while the network fed with GLCM features misclassified all the samples from this category. In addition, both the networks (fed with GLCM and LBP features) failed to predict any sample correctly from medium faulty class. It happensdue to less representation of this class for training and similarly less in the test data (16 to be exact).

#### 6.1.6. Multiclass Classification Using Augmented Data

After augmentation, both networks are able to predict a few samples from each class, as shown in Table 8. LBP features achieved 3.8% higher accuracy as compared to their previous preaugmented data case, while the prediction accuracy associated with GLCM features dropped by 1.8% overall after augmentation. This drop in accuracy is due to comparatively high generalization error for the healthy class after augmentation. The optimized ANN architecture for postaugmented multiclass classification ended up having three hidden layers with [30-20-10] hidden neurons and [30-20-20] hidden neurons for GLCM and LBP features, respectively. Overall, the LBP features obtained superior classification accuracy and proved to be a better hand-crafted feature choice with ANN, both for binary and the multiclass classification tasks. Moreover, the optimized ANN architectures fed with LBP features ended up having fewer hidden layers as compared to the optimized architectures for GLCM features' case. Concluding, the LBP features outperformed the GLCM features with respect to both accuracy and the network computational complexity.

### 6.2. Convolutional Neural Network Results

In this section, binary and multiclass classification is presented using customized CNN architectures. Considering the ANN results presented in the previous section, it is clear that the classifier's performance both for binary and multiclass classification was improved after data augmentation. This is because the original data have limited as well as an imbalanced representation of target classes; hence, the network shows better performance with the augmented data. Based on that, we consider only postaugmented data for CNN training. This section will explain the design of task-specific optimized CNN architectures and the obtained results.

To estimate the CNN architecture for a specific task like binary classification, it is started from scratch with a single convolutional layer with a pooling and a fully connected layer. The complexity is increased progressively until convergence is achieved for the specific task based on the training data results. The minimum filter size for convolutional layer is chosen 3 × 3 to start with and increased to be 5 × 5 and up to 7 × 7 maximum. All the filters are applied with stride 1. The combinations of different filter sizes are tested in the convolutional layer. To estimate the number of filters to be used per convolutional layer, initially 8 filters were chosen and further increased by a multiple of 2. Similarly, the number of convolutional layers is increased to achieve the improved results. The optimized number of convolutional layers is estimated based on best training data results. In the pooling layer, max-pool scheme is opted for to reduce the size with stride 2. After pooling layer, the fully connected layer is added with the Softmax activation function. In estimating the optimal CNN architecture, a large number of architectures were tested with different choices of convolutional layers, convolutional filter size, and the number of pooling layers. During the process, both the accuracy of the network as well as the computational cost is observed. Therefore, by observing the trade-off between accuracy and computational complexity, the best CNN architecture is selected. Since three separate classification tasks are performed in this research, three independent, task-specific, and customized CNN models are estimated.

**Figure 6:**
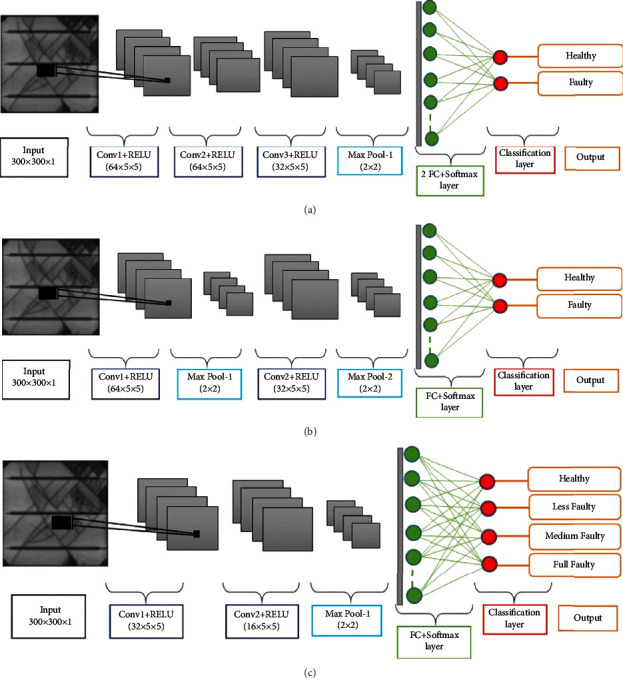
Estimated architectures of customized CNN for different classification tasks. (a) Binary classification. (b) Binary classification with 0.5 as threshold. (c) Multiclass classification.

We develop the customized CNN model for each of the classification problems: binary classification, binary classification with 0.5 as threshold, and multiclassification. A total of 25 different architectures of CNN are tested with a suitable selection of hyperparameters to find the optimized network for the three classification tasks. The final, customized architecture for each of the classification tasks is presented in Figure 6. The optimized CNN architecture for binary classification has the following specifications: three convolutional layers with ReLU (Rectified Linear Unit) activation function; the optimum filter size is 5 × 5 in all layers; the first and second convolutional layers have 64 filters, while the third layer has 32 filters. The convolutional layers are followed by a single pooling layer with max pooling criteria applied with stride 2. Next, two fully connected (FC) layers are added, and finally the softmax function is used for prediction. For the task of binary classification with 0.5 as threshold, the optimized CNN architecture has Conv-Pool-Conv-Pool-FC layers arrangement, where the first convolutional layer has 64 and second layer has 32 filters, both with filter size of 5 × 5. The CNN estimated for multiclass classification has Conv-Conv-Pool-FC layer architecture with 32 and 16 filters in the first and second convolutional layers, respectively. In all the estimated CNN architectures, the size of the FC layer is kept at 32 neurons, since it is observed as a suitable minimum size for the FC layer. The training options and the hyperparameters for customized CNNs are summarized in Table 9.

Now we present the classification results of the estimated CNN models on the test data shown in Table 10. The CNN developed for binary classification achieved 94.3% accuracy. On comparing with the binary classification case, the CNN showed 2.1% higher accuracy than the best ANN model-based results, achieved with LBP features (shown in Table 4). Considering the classwise accuracy, the CNN achieved 0.4% and 5.6% higher accuracy for the healthy and faulty classes, respectively. Concluding, to detect a faulty PV cell, the CNN is 5.6% more accurate than the ANN-based model.

In the second task, binary classification with 0.5 as the threshold, the developed CNN achieved 89.3% accuracy, which is 4.5% higher than the deep ANN model for the same task. Similarly, looking at the classwise results, there is a marginal improvement of 1.4% in accuracy for the healthy class. However, the CNN outperforms the ANN with a margin of 11.4% for the faulty class. Since the detection of a faulty cell is more important than that of the healthy cell, the CNN does the job with significantly improved accuracy.

Finally, there is an improvement of 7.4% overall for multiclass classification with customized CNN than that of the ANN model (see Table 8 (b)). By carefully observing the Table 10 (c), it is evident that the CNN confused the majority of misclassified samples with the nearest class. For instance, 19 samples of the healthy class were confused with less faulty class. Hence, most of the misclassified samples were confused with the nearest class. This happened for each of the target class. In contrast, the ANN confused majority of the samples with the far class, e.g., 30 healthy samples were misclassified as full faulty samples, as shown in Table 8 (b). Therefore, it can be concluded that the CNN not only obtained better results quantitatively but also qualitatively.

**Figure 7:**
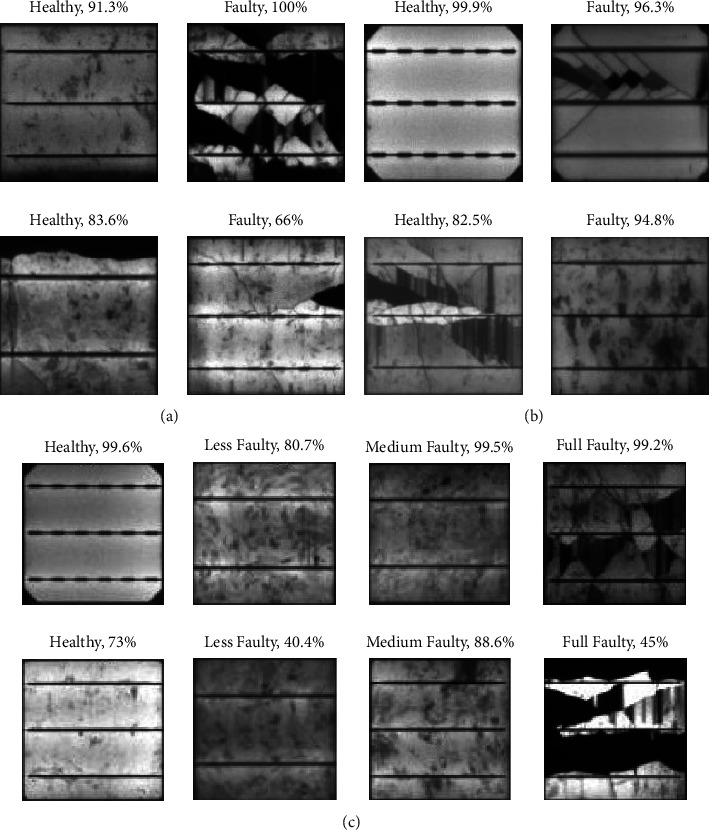
Qualitative results of proposed customized CNN on random test data samples with confidence percentage. (a) Binary classification. (b) Binary classification with 0.5 as threshold. (c) Multiclass classification.

Figure 7 shows the classification results of a few random samples from test data with a confidence percentage, for the different classification tasks. It is worth mentioning that the confidence percentage does not reflect the defect probability predicted by CNN. For instance, in Figure 7(a), the CNN correctly predicted a healthy sample (top left) with a confidence percentage of 91.3%. This means the network finds that 91.3% of the content of the image matches to the foot print, which it has learned for the healthy class, and it is represented as confidence in its decision. However, the high confidence does not necessarily mean that the prediction is correct. This is equally possible that the network misclassifies even with a higher percentage. This is also the case in the CNN results, as the faulty sample in bottom left of Figure 7(a) is misclassified as healthy. Similarly, the samples shown in the bottom row of Figure 7(b) are both misclassified by the network. For multi-classification prediction, the CNN confused the less faulty class and the medium faulty class with each other and therefore, both samples from these classes in the bottom row of Figure 7(c) were misclassified.

Overall, the CNN model outperformed all the ANN models for each of the classification tasks. In addition to achieving the best classification accuracy, the developed CNN models also confirm the real-time classification of EL image data. This is because the developed CNN models are lighter and computationally less expensive. The models are developed from scratch adopting the bottom-up approach for estimating the architectural depth while considering the complexity-accuracy tradeoff, in contrast to choosing a pretrained CNN and progressively reducing the size of the classification task in hand like in few existing studies. The deep learning approach proved to be a good approach since the deep ANN models with hand-crafted features also achieved comparative accuracy for binary classification task.

## 7. Comparison with Existing Studies

In this section, a comparative analysis with existing studies which specifically used the same EL image database is presented.

### 7.1. Binary Classification

#### 7.1.1. Classification Accuracy

The authors in the study [38] performed data augmentation and presented the two-way binary classification, similar to the two strategies for binary classification employed in this study. In this subsection, a comparison of results for binary classification (using data of two classes: healthy prob. 0.0 and full faulty) is presented, while the comparison for binary classification with 0.5 threshold is discussed later in the subsection 5.2. For binary classification, the author [38] claimed 82.44% accuracy using SVM classifier, while the best results using the proposed methods are achieved at 92.2% and 94.3% accuracy with ANN and CNN, respectively. Hence, the proposed techniques produced 11.86% higher accuracy in comparison to [38]. In the other study [40], the authors presented a VGG structure-based CNN classifier for binary classification of EL image data and achieved the best accuracy of 93.5%, while the four-fold average accuracy of 93.02%. In contrast, the accuracy achieved using the proposed method is recorded of 94.3%, which is 0.8% (slightly) higher. Concretely, the proposed CNN-based customized model outperforms the state-of-the-art results for binary classification of the EL image data in terms of accuracy.

#### 7.1.2. Significance of Hand-Crafted Features

The authors of the study [38] extracted scale-invariant feature transform (SIFT), speed up robust features (SURF), KAZE, and histogram of oriented gradients (HOG) features from images as well as combinational features using dense sampling. In general, such features are suitable and primarily used for object detection and classification tasks where the nature of complexity as well as the number of classes is much higher. In comparison, a less computationally expensive features: GLCM and LBP are proposed in this study, and yet obtained better classification results.

#### 7.1.3. Classifier's Computational Complexity

The VGG structure-based 6-layered CNN architecture, including four convolutional layers, was presented in the study [40] for binary classification of EL image data. The author split the data into 80 : 20 ratios for training and testing purposes, respectively, and achieved the best accuracy as 93.5%, while the four-fold average accuracy was 93.02%. In comparison, a customized yet lighter CNN architecture having three convolutional layers is proposed which achieves 94.3% accuracy. Considering the computational cost, the CNN presented model in the study [40] had 2,410,208 parameters, whereas the proposed model has 1,331,264 parameters, which was almost half.

#### 7.1.4. Data Augmentation

The original dataset contains a total of 2,624 EL images. The authors of the study [38] performed data augmentation to increase the image samples and obtained a total of 196,800 samples after augmentation. In comparison, in this research a total of 6000 samples are prepared after augmentation, which is almost 39× less in size than [38]. In the study [40], the author performed data augmentation to balance the classes; however, no information was provided regarding data size after augmentation.

#### 7.1.5. Processing Time for Training and Testing

Considering the processing time, the proposed ANN-based classifier elapsed 296 sec for training (using 6,000 samples) and 7.4 sec to classify the test data (using 394 image samples) with an accuracy of 92.12%. Hence, 18.78 msec of time to classify individual test images, reflects the real-time processing speed. Moreover, the proposed CNN-based classifier elapsed 34 min 52 sec for training and 37.2 sec for prediction of test data, making 94.4 msec to classify the individual test data sample. The authors in the study [40] claimed 8.07 msec to predict the individual test image sample; however, the comparison for prediction time is not straight forward.

Firstly, the hardware used in this research has the following specifications: Intel core i3 with 2.4 GHz processor and 2 GB RAM, which are low as compared to the hardware specification (Intel core i5 with 3.2 GHz processor, RAM not specified) reported in the study [40]. Another reason for the higher accumulated processing time in this research is the size of the image. In the proposed work, the original size of the image sample is used, i.e., 300 × 300 pixels, while the author in the study [40] resized (down sampled) the image to 100 × 100 pixels before use, which makes the image size 9× smaller than the original size and therefore reduces the processing time. Although the processing time to predict the health of a test sample in this study is larger under the described hardware constraint, it still ensures the real-time grading of PV cells with higher accuracy. Comparing the convergence time, the training time for the CNN used in the study [40] is reported 13 hr 45 min, whereas the proposed model took only 35 min for training, which is 23× less time for convergence.

### 7.2. Binary Classification with 0.5 as Threshold

For the case of binary classification with 0.5 as the threshold, the author of the study [38] employed CNN with transfer learning and achieved an accuracy of 88.4%, whereas the proposed CNN model achieved an accuracy of 89.3%. The improvement in accuracy is minor; however, the architectural complexity of the proposed model is much lower. The VGG-19 model with 14 convolutional layers and a large number of filters used in [38] made it much more computationally complex than the proposed CNN model, which has only two convolutional layers, one pooling and one fully connected layer. Moreover, the training data after augmentation is much smaller which makes the proposed customized CNN architecture much more efficient. In addition, the study [38] employs GPU for the experiment, making it a hardware-demanding solution. In contrast, the proposed CNN-based classifier works in real-time on a CPU machine and yet achieves better accuracy. The training time elapsed by the proposed CNN-based model for this particular task of binary classification is 47 min 6 sec, and the test time is 31.6 sec. Therefore, the prediction for a single test data sample is carried out in 80.2 msec making the proposed classifier suitable for real-time classification of PV cells.

### 7.3. Multiclass Classification

In addition to the two kinds of binary classifications, the multiclass classification of EL image data is presented. The best overall accuracy of 76.1% is achieved by the proposed deep feed-forward neural network with LBP features, while the customized CNN-based classifier achieves 83.5% accuracy overall. The testing time for multi-classification using the proposed customized CNN is recorded as 28.7 sec for 394 samples. In other words, it took 72.84 msec to predict the health of a single image sample of a PV cell, making the proposed classifier a real-time suitable solution to perform multi-classification on a CPU machine. The multiclassification of EL image data has not been presented in existing literature yet; therefore, the state-of-the-art results are presented in this category.

The summary of comparative analysis is presented in Table 11. The results show the significance of the proposed methods over the existing studies both quantitatively and computationally.

## 8. Conclusions

In this study, the fault-level binary and multiclassification of EL image data are presented. The deep ANN models with a minimum suitable size are estimated and optimized, where hand-crafted features are extracted from the image data. The estimated deep architectures of ANN show best performance when fed with LBP hand-crafted features, achieving 92.1% and 76.1% accuracy for binary and multiclass classification, respectively. In addition to ANN, customized, task-oriented, and light-weight models of CNN are developed. The proposed CNN-based customized model achieved the state-of-the-art 94.3% classification accuracy for the binary classification. The proposed model achieved 83.5% state-of-the-art accuracy for multiclass classification as well as employing a CNN-based model. In comparison, the proposed models achieved enhanced performance than the existing solutions, both quantitatively and computationally. The proposed solution may be used for real-time health assessment of PV solar cells using EL imaging. The results also support the effectiveness of CNN-based approach for real time image-based PV cell health classification. Considering the limitation, the data was balanced to equalize the number of samples by augmentation and while this procedure, the number of augmented images were at a different scale with respect to number. In future, the advance methods for image augmentation may be used such as the general adversarial network (GAN) to produce high-quality augmented samples for better data representation and improved network learning.

## Figures and Tables

**Table 1:** Details of the dataset used in the proposed work.

Dataset detail	
Number of images	2624 images
Image size	300 × 300
Image type	Gray scale
Bits/image	8-bit
Number of solar modules	44 modules (18 monocrystalline + 26 polycrystalline)
Target classes	4 (defect probability: 0, 0.33, 0.66, 1)

**Table 2:** Size of electroluminescence image data: classwise.

Class	Label	Defect probability	Number of images
1	Healthy	0.00	1508
2	Less faulty	0.33	295
3	Medium faulty	0.66	106
4	Faulty	1.00	715

**Table 3:** Binary classification results of deep ANN using original data: (a) GLCM features and (b) LBP features.

		Actual class	
		Healthy	Full faulty
*(a) GLCM features*			
Predicted class	Healthy	178	32
	Full faulty	48	75
Classwise accuracy		78.7%	70.1%
Overall accuracy		75.9%	

*(b) LBP features*			
Predicted class	Healthy	178	32
	Full faulty	48	75
Class-wise accuracy		78.7%	70.1%
Overall accuracy		75.9%	

**Table 4:** Binary classification results of deep ANN using augmented data: (a) GLCM features and (b) LBP features.

		Actual class	
		Healthy	Full faulty
*(a) GLCM features*			
Predicted class	Healthy	203	24
	Full faulty	23	83
Classwise accuracy		89.8%	77.6%
Overall accuracy		85.9%	

*(b) LBP features*			
Predicted class	Healthy	216	16
	Full faulty	10	91
Classwise accuracy		95.6%	85%
Overall accuracy		92.2%	

**Table 5:** Binary classification results of deep ANN with 0.5 as threshold using original data: (a) GLCM features and (b) LBP features.

		Actual class	
		Healthy	Full faulty
*(a) GLCM features*			
Predicted class	Healthy	223	33
	Full faulty	48	90
Classwise accuracy		82.3%	73.2%
Overall accuracy		79.4%	

*(b) LBP features*			
Predicted class	Healthy	230	29
	Full faulty	41	94
Classwise accuracy		84.9%	76.4%
Overall accuracy		82.2%	

**Table 6:** Binary classification results of deep ANN with 0.5 as threshold using augmented data: (a) GLCM features and (b) LBP features.

		Actual class	
		Healthy	Full faulty
*(a) GLCM features*			
Predicted class	Healthy	249	38
	Full faulty	22	85
Classwise accuracy		91.9%	69.1%
Overall accuracy		84.8%	

*(b) LBP features*			
Predicted class	Healthy	247	38
	Full faulty	24	85
Classwise accuracy		91.1%	69.1%
Overall accuracy		84.3%	

**Table 7:** Multiclass classification results of the deep ANN using original data: (a) GLCM features and (b) LBP features.

		Actual class			
		Healthy	Less faulty	Medium faulty	Full faulty
*(a) GLCM features*					
Predicted class	Healthy	194	39	12	47
	Less faulty	0	0	0	0
	Medium faulty	0	0	0	0
	Full faulty	32	6	4	60
Classwise accuracy		85.8%	0%	0%	56.1%
Overall accuracy		64.5%			

*(b) LBP features*					
Predicted class	Healthy	196	18	6	34
	Less faulty	0	21	8	5
	Medium faulty	0	0	0	0
	Full faulty	30	6	2	68
Classwise accuracy		86.7%	46.7%	0%	63.5%
Overall accuracy		72.3%			

**Table 8:** Multiclass classification results of the deep ANN using augmented data: (a) GLCM features and (b) LBP features.

		Actual class			
		Healthy	Prob. 0.33	Medium faulty	Full faulty
*(a) GLCM features*					
Predicted class	Healthy	148	13	3	20
	Prob. 0.33	27	27	02	9
	Medium faulty	12	2	9	15
	Full faulty	39	3	2	63
Classwise accuracy		65.5%	60%	56.2%	58.9%
Overall accuracy		62.7%			

*(b) LBP features*					
Predicted class	Healthy	182	7	2	25
	Less faulty	7	34	3	2
	Medium faulty	7	2	10	6
	Full faulty	30	2	1	74
Classwise accuracy		80.5%	75.5%	62.5%	69.1%
Overall accuracy		76.1%			

**Table 9:** Training options selected for the training of customized CNN for all three types of classifications of the PV dataset.

Training options	Binary classification	Binary classification with 0.5 as threshold	Multi-classification
Min batch size	10	5	15
Number of epochs	3	3	3
Validation frequency	3 iterations	3 iterations	3 iterations
Validation patience	∞	∞	∞
Initial learning rate	0.0001	0.0001	0.0001
Number of iterations	75	90	90

**Table 10:** The customized CNN classification results: (a) binary classification, (b) binary classification with 0.5 as threshold, and (c) multiclass classification.

		Actual class			
		Healthy		Full faulty	
*(a) Binary classification*					
Predicted class	Healthy	249		38	
	Full faulty	22		85	
Classwise accuracy		91.9%		69.1%	
Overall accuracy		84.8%			

*(b) Binary classification with 0.5 as threshold*					
Predicted class	Healthy	247		38	
	Full faulty	24		85	
Classwise accuracy		91.1%		69.1%	
Overall accuracy		84.3%			

*(c) Multi-class classification*					
		*Healthy*	*Less faulty*	*Medium faulty*	*Full faulty*
Predicted class	Healthy	194	4	1	4
	Less faulty	19	38	1	7
	Medium faulty	6	1	12	11
	Full faulty	7	2	2	85
Classwise accuracy		85.8%	84.4%	75%	79.4%
Overall accuracy		83.5%			

**Table 11:** Comparative analysis for electroluminescence image database classification.

Comparison parameters	Study				
	[38]		[40]	Proposed method	
Database (original)	2,624		2,624	2,624	
Data division (%)	75–25 (train-test)		80–20 (train-test)	70-15-15 (train-val-test)	
Training samples after augmentation	196,800		Not mentioned	6,000	
Features	SIFT, SURT, KAZE, HOG, PHOW		×	GLCM, LBP	×
Classifier	SVM	CNN	VGG-based CNN	Deep ANN	Customized CNN
Binary classification accuracy	82.44%	×	93.02%	92.1%	94.3%
Binary classification accuracy with 0.5 as threshold	×	88.42%	×	84.8%	89.3%
Multiclassification accuracy	×	×	×	76.1%	83.5%

## Data Availability

The datasets are available from the corresponding author upon request (https://github.com/zae-bayern/elpv-dataset).
